# Readiness, barriers, and attitude of students towards online medical education amidst COVID-19 pandemic: A study among medical students of Ebonyi State University Abakaliki, Nigeria

**DOI:** 10.1371/journal.pone.0284980

**Published:** 2023-04-27

**Authors:** Edmund Ndudi Ossai, Irene Ifeyinwa Eze, Chukwuma David Umeokonkwo, Chukwuemeka Obioma Izuagba, Lawrence Ulu Ogbonnaya

**Affiliations:** 1 Department of Community Medicine, Ebonyi State University Abakaliki, Abakaliki, Nigeria; 2 Department of Community Medicine, Alex Ekwueme Federal University Teaching Hospital Abakaliki, Abakaliki, Nigeria; University of Science and Technology of Fujairah, YEMEN

## Abstract

**Introduction:**

The COVID-19 pandemic caused massive disruption to medical education in Nigeria, necessitating the call for online medical education in the country. This study assessed the readiness, barriers, and attitude of medical students of Ebonyi State University Abakaliki, Nigeria, to online medical education.

**Methods:**

A cross-sectional study design was employed. All matriculated medical students of the university participated in the study. Information was obtained using a pre-tested, semi-structured questionnaire which was self-administered. Good attitude towards information and communication technology (ICT) based medical education was determined by the proportion of respondents correctly answering 60% of nine variables. Readiness for online classes was determined by the proportion of students who preferred either a combination of physical and online lectures or only online medical education amidst the COVID-19 pandemic. Chi-square test and multivariate analysis using binary logistic regression analysis were used in the study. A p-value of <0.05 determined the level of statistical significance.

**Results:**

Four hundred and forty-three students participated in the study (response rate; 73.3%). The mean age of the students was 23.0±3.2 years. The majority of the respondents, 52.4%, were males. The students’ most preferred sources for studying before the COVID-19 pandemic included textbooks, 55.1% and lecture notes, 19.0%. The commonly visited websites included Google, 75.2%, WhatsApp, 70.0% and YouTube, 59.1%. Less than half, 41.1%, have a functional laptop. The majority, 96.4%, have a functioning email address, while 33.2% participated in a webinar during the COVID-19 pandemic. Though 59.2% had a good attitude towards online medical education, only 56.0% expressed readiness for online medical education. The major barriers to online medical education included poor internet connectivity, 27.1%, poor e-learning infrastructure, 12.9% and students not having laptops, 8.6%. Predictors of readiness for online medical education included previous participation in a webinar, AOR = 2.1, (95%CI: 1.3–3.2) and having a good attitude towards IT-based medical education, AOR = 3.5, (95%CI: 2.3–5.2).

**Conclusions:**

The majority of the students showed readiness for online medical education. Lessons from COVID-19 pandemic necessitate the initiation of online medical education. University authorities should ensure that every enrolled medical student owns or have access to a dedicated laptop through a university-mediated arrangement. Adequate attention should be given to the development of e-learning infrastructure, including steady internet services within the confines of the university.

## Introduction

Worldwide, the COVID-19 pandemic has affected all sectors, including the educational sector [1]. The pandemic created an enormous disruption of education systems in history, involving nearly 1.6 billion learners in more than 190 countries and continents [2], adversely affecting students’ academic expectations in the sub-Saharan Africa [3–5]. The fast spread of COVID-19 brought about many changes in the educational sector, including restrictions on movement and lockdowns of institutions of learning [6, 7]. In Nigeria, the COVID-19 pandemic impacted higher institutions following the lockdown in many ways: disruption of the academic calendar, teaching and learning gap, loss of workforce, reduction of international education, and cancellation of local and international conferences [8]. Furthermore, the coronavirus pandemic exposed massive socio-economic inequality and learning disparity in Nigeria’s education system, with wealthy families sending their children to private schools equipped for remote learning in contrast to the poor public school facilities [9].

The educational landscape is undergoing a radical transformation to cushion the effect of the pandemic and revamp the education system, including shifting toward the e-learning [10, 11]. E-learning can enhance knowledge efficiency through easy access to a large amount of information within the global village, promoting personal knowledge accumulation and group knowledge sharing [12, 13]. Furthermore, e-learning increases satisfaction and decreases stress by enabling students and lecturers to participate in class in their comfort zone and is cost-effective as it reduces travel time and infrastructural development in terms of buildings [14, 15].

Even with the numerous benefits of e-learning, developing countries, including Nigeria, are still challenged to shift from the traditional teaching method to e-learning during the pandemic [16]. The challenges arise from varying degrees of preparedness of the institutions, including the inadequate skill of lecturers [16], social inequalities [1] and lack of infrastructures, irregular power supply, high cost of internet data services and personal computer (PC) and Laptop, poor internet connectivity [17], all of which threaten its applicability. Furthermore, although around 83% of Nigerians have mobile phone connections, the proportion is skewed towards the few high socio-economic and urban households, most of whom are private school students with a learning advantage over their public school peers [9]. Therefore, assessing the barriers to e-learning in terms of the availability of digital facilities, including the adequacy in quantity and quality, can help academic administrators create mechanisms that can enhance users’ attitudes and readiness for adopting the e-learning platform.

E-learning system has become the most preferred learning platform during global pandemic periods such as COVID-19, where movement is restricted [10, 11]. However, there needs to be a more empirical examination of the factors underlying the adoption of e-learning systems. Factors such as perceived usefulness of e-learning, ease of use, pressure to use, good technical skills and the availability of resources needed to use e-learning impact students’ attitudes and readiness towards the e-learning [18, 19]. Yet, assessment of the user characteristics in e-learning systems, especially in a developing country like Nigeria, appears to be limited, despite being a prerequisite to introducing successful e-learning systems [18]. Successful system implementation and adoption by learners requires a solid understanding of user acceptance processes with these technologies. Assessing the attitude is vital in analysing consumer readiness to adopt, as a favourable attitude shows a greater probability that learners will be ready to accept the new learning system [19, 20]. Acceptance of the new learning system will be worthwhile as earlier studies reported enhanced knowledge efficiency [12, 13] and mean performance of users in favour of online education compared to the same course delivered face to face [21]. Since users’ views tend to impact the advancement of the e-learning [18, 19], understanding how students perceive and react to elements of e-learning and factors that influence readiness for effective e-learning is essential and timely. This study aimed to ascertain the barriers, attitudes, and readiness for adopting e-learning among medical students in a Nigerian university.

## Methods

### Description of the study area

The College of Health Sciences of Ebonyi State University Abakaliki, Nigeria, was established in 1999, the same year the university was established. Based on the recommendation of the Nigerian Medical and Dental Council, the university admits an average of 100 medical students every year. The study of Medicine in Nigeria runs for six years, each year being recognized as a level. The first year of study (also called 100 level) is called the preliminary year, and during this period, academic activities take place at the Faculty of Science of the University. The second and third years of study are the pre-clinical training period, while the fourth to sixth year is the clinical training period. The university’s medical students receive clinical training at Alex Ekwueme Federal University Teaching Hospital Abakaliki, Nigeria.

#### Impact of COVID-19 pandemic on medical education

The university, including the medical school, was closed down by the Nigeria Government during the peak of the COVID-19 pandemic on 19^th^ March 2020; after the World Health Organization declared the COVID-19 outbreak a global pandemic on 11th March 2020. The closure involved all higher institutions in the country and aimed to curtail the spread of the virus. Like many public universities in Nigeria, the university did not initiate any form of online learning during the period the university was closed down. The study occurred between September and October 2021, after the university was re-opened in February 2021 for academic activities following the COVID-19 lockdown.

### Study design and study population

A cross-sectional design was employed. The Strengthening the Reporting of Observational Studies in Epidemiology (STROBE) was used to construct and conduct the study. All matriculated medical students at the university were included in the study. The students who were not present on the days of data collection and those who refused to consent to participate were excluded from the study. A total of four hundred and forty-three (443) medical students participated in the study representing a response rate of 73.8%.

### Study instrument

A pre-tested, semi-structured, self-administered questionnaire that the researchers designed after a review of some literature [8, 13] was used for data collection. The questionnaire was pretested on 45 medical students (10% of the study population) at another university.

### Data management

Data entry and analysis were done using International Business Machine (IBM) Statistical Package for Social Sciences (SPSS), version 25. Chi-square test and multivariate analysis using binary logistic regression analysis were used in the research, and a p-value of <0.05 determined the level of statistical significance. The outcome measure of the study was the readiness of the medical students to commence online medical education at the university. This was represented by the proportion of students who preferred in-class and e-learning approaches and e-learning methods only in response to how to continue medical education amidst the COVID-19 pandemic. Furthermore, a good attitude towards information and communication technology (ICT) based medical education was determined by the proportion of respondents correctly answering 60% of nine variables. In determining the predictors of the readiness of the students to commence online medical education, variables with a p-value of <0.2 on bivariate analysis were included in the binary logistic regression model. The binary logistic regression analysis results were presented using an adjusted odds ratio (AOR) and 95% confidence interval. A p-value of <0.05 determined the statistical significance level.

### Ethical consideration

Ethical approval for the study was obtained from the Research and Ethics Committee of Ebonyi State University Abakaliki, Nigeria. (Reference number, EBSU/DRIC/UREC/Vol.04/064). The students signed a written informed consent form before participating in the study. The nature of the research and their involvement were known to them. The students were informed that participation in the survey was voluntary and assured that all information provided through the questionnaire would be kept confidential.

## Results

Table 1 shows the socio-demographic characteristics of the respondents. Four hundred and forty-three students participated in the study (response rate; 73.3%). The mean age of the students was 23.0±3.2 years. The majority of the respondents, 52.4%, were males and single 428 (96.9%). Most students were in 300 academic level 102(23.0%) and 400 academic level 103(23.3%).

**Table 1:** Socio-demographic characteristics of respondents.

Variable	(n = 443) Frequency (%)
**Age of respondents**	
Mean±(SD)	23.0±3.2
**Age of respondents in groups**	
<20 years	37 (8.4)
20–24 years	291 (65.7)
25–29 years	99 (22.3)
≥30 years	16 (3.6)
**Gender**	
Male	232 (52.4)
Female	211(47.6)
**Academic level**	
100 level	23 (5.2)
200 level	93 (21.0)
300 level	102 (23.0)
400 level	103 (23.3)
500 level	64 (14.4)
600 level	58 (13.1)
**Marital status**	
Single	428 (96.6)
Married	15 (3.4)
**Religion of respondent**	
Christianity	437 (98.6)
Islam	5 (1.2)
Traditional religion	1 (0.2)
**Ethnicity of respondent**	
Igbo	424 (95.7)
Others**	19 (4.3)
**Educational attainment of Father**	
No formal education	28 (6.3)
Primary education	48 (10.8)
Secondary education	82 (18.5)
Tertiary education	285 (64.3)
**Educational attainment of Mother**	
No formal education	28 (6.3)
Primary education	45 (10.2)
Secondary education	77 (17.4)
Tertiary education	293 (66.1)

Table 2 shows access to and application of internet services among the respondents. Majority of the respondent had an android phone 373 (84.2%2), a functional e-mail address 427 (96.4%) and access to the internet 414 (93.5%). Less than half, 184 (41.1%), had a functional laptop, and 147 (33.25) participated in webinars during the pandemic. The most preferred source for studying before the COVID-19 pandemic were textbooks 224(55.1%) and lecture notes 84(19.0%). The commonly visited websites included google 333 (75.2%), WhatsApp 310 (70%), and YouTube 262 (59.1%). School-based activities that the internet was mostly used were course registration 362 (81.7%) and payment of school fees 327(73.8%).

**Table 2:** Access to and application of internet services among the respondents.

Variable	(n = 443) Frequency (%)
**Respondent has a functional laptop**	
Yes	182 (41.1)
**Ownership of devices**	
Android phone	373 (84.2)
Tablet	49 (11.1)
iphone	46 (10.4)
Internet modem	46 (10.4)
**Respondent has a functional email address**	
Yes	427 (96.4)
**Respondent has access to the internet**	
Yes	414 (93.5)
**Respondent participated in Webinar during the pandemic**	
Yes	147 (33.2)
**The most preferred source for studying in medical school before COVID-19** **	
Textbooks	244 (55.1)
Lecture notes	84 (19.0)
E-textbooks	65 (14.7)
Internet	39 (8.8)
Uncertain	11 (2.5)
**Commonly visited websites** **	
Google	333 (75.2)
WhatsApp	310 (70.0)
YouTube	262 (59.1)
Facebooks	164 (37.0)
Instagram	127 (28.7)
Twitter	114 (25.7)
Google scholar	40 (9.0)
Yahoo	39 (8.8)
**School-based activities that use the Internet before COVID-19** **	
Course registration	362 (81.7)
Payment of school fees	327 (73.8)
Checking results after examination	301 (67.9)
Further reading after lectures	242 (54.6)
Research work/projects	227 (51.2)
Registration for examinations	224 (50.6)
Submission of assignments	123 (27.8)
Lectures	36 (8.1)

**multiple responses encouraged

Table 3 shows readiness to online medical education. Majority of the respondents, 235 (53.3%), expressed readiness for a combination of in-class and e-learning approaches to medical education amidst the Covid-19 pandemic. Only 12 (2.7%) preferred e-learning methods only.

**Table 3:** Readiness for online medical education among the respondents.

Variable	(n = 443) Frequency
**Approach to medical education amidst the COVID-19 pandemic**	
Physical learning only	184 (41.5)
In-class and e-learning approaches	235 (53.3)
Rely on e-learning methods only	12 (2.7)
Uncertain	11 (2.5)
**Readiness for online medical education**	
Yes	248 (56.0)
No	195 (44.0)

Table 4 shows barriers to online medical education. The major barriers to online medical education included poor internet connectivity, 120 (27.1%), poor e-learning infrastructure, 57(12.9%), and students not having laptops, 38(8.6%). The majority of the respondents, 275(62.1%), reported that the university has no functional virtual library.

**Table 4:** Barriers for online medical education among the respondents.

Variable	(n = 443) Frequency (%)
**Approach to medical education amidst the COVID-19 pandemic**	
Physical learning only	184 (41.5)
In-class and e-learning approaches	235 (53.3)
Rely on e-learning methods only	12 (2.7)
Uncertain	11 (2.5)
**Readiness for online medical education**	
Yes	248 (56.0)
No	195 (44.0)
**The main barrier to e-learning application in medical school**	
Poor internet connectivity	120 (27.1)
Poor e-learning infrastructure	57 (12.9)
Students do not have laptops	38 (8.6)
High demand on time	36 (8.1)
Lack of technical skills on the part of students	33 (7.4)
Cost of internet services	31 (7.0)
Inadequate preparation for medical practice	21 (4.7)
Reduction in the interaction between teachers and medical students	18 (4.1)
Poor preparation of medical students for examinations	13 (2.9)
No response	15 (3.4)
**Need to increase school fees because of the introduction of e-learning**	
Yes	24 (5.4)
No	419 (94.6)
**University has a functional virtual library**	
Yes	66 (14.9)
No	275 (62.1)
Don’t know	102 (23.0)

Table 5 shows the attitude towards information and technology-based medical education. Overall good attitude towards online medical education was noted in 263 (59.4%) respondents. However, 68.8% of the respondents viewed that lectures for medical teaching should be made available on the university e-learning portal, and 60.9% opined that online learning platforms should complement in-class teachings in medical school.

**Table 5:** Attitude towards information & communication technology (ICT) based medical education.

Variable	(n = 443) Frequency (%)
Lectures for medical teaching should be made available on the university e-learning portal	305 (68.8)
Physical lectures only should continually be used for medical education	232 (52.4)
Every medical student should have a functional laptop	204 (46.0)
Online learning platforms should be used to complement in-class teachings in medical school	270 (60.9)
There is no need for the use of computers in medical education	255 (57.6)
Need for training students in e-learning content development	302 (68.2)
Adoption of e-learning could increase the satisfaction of medical students with training	290 (65.5)
E-learning could improve the quality of learning in a medical school	299 (67.5)
Need to improve e-learning infrastructure in medical school	330 (74.5)
**Attitude towards ICT-based medical education**	
Good	263 (59.4)
Poor	180 (40.6)

(Only correct responses indicated on the table)

Table 6 shows predictors of readiness for online medical education. The predictors of readiness for online medical education included previous participation in a webinar, AOR = 2.1, (95%CI: 1.3–3.2) and having a good attitude towards ICT-based medical education, AOR = 3.5, (95%CI: 2.3–5.2).

**Table 6:** Predictors of readiness for online medical education among the respondents.

Variable	Readiness for online lectures (n = 443)	p-value	Adjusted odds ratio	95%confidence interval
	Frequency (%)			
**Age of respondents in groups**				
<20 years	21 (56.8)	0.761	NA	
20–24 years	166 (57.0)			
≥25 years	61 (53.0)			
**Gender**				
Male	138 (59.5)	0.120	1.307	0.873–1.956
Female	110 (52.1)		1	
**Period of training**				
Non-clinical students	126 (57.8)	0.448	NA	
Clinical students	122 (54.2)			
**Marital status**				
Single	243 (56.8)	0.072	2.096	0.632–6.951
Married	5 (33.3)		1	
**Have a functional laptop**				
Yes	107 (57.5)	0.577	NA	
No	141 (54.9)			
**Participation in Webinar during the COVID-19 pandemic**				
Yes	102 (69.4)	<0.00	2.080	1.343–3.222
No	146 (49.3)		1	
**Attitude towards ICT-based med. education**				
Good	181 (68.8)	<0.001	3.459	2.303–5.195
Poor	67 (37.2)		1	

ICT Information & communication technology NA Not applicable

## Discussions

The study found that students’ major barriers to online medical education included poor internet connectivity, poor e-learning infrastructure, unavailability of personal laptops, and lack of functional virtual libraries in the university. This finding is consistent with the result of earlier studies on assessing the availability and utilisation of ICT resources in teaching in Nigeria, which revealed that the availability of digital resources for effective instructional delivery in schools is relatively low [22, 23]. Under such situations, even if teachers are well trained, make themselves and their instructional materials available online, and are willing to impart knowledge to the students, they are hindered by the lack or inadequacy of the technological equipment and facilities [10]. This is because, many students lack the means to access online materials from home due to poor connectivity or the unavailability of personal laptops [10]. The finding aligns with a previous study which noted that nearly half of the low-income families lacked sufficient ICT devices at home [10]. This research revealed the need for adequate availability of digital resources amidst and post covid-19 era in Nigerian Universities. This view is supported by an earlier study which showed a smoother and positive shift for schools which provided adequate resources for students and academic staff [9].

The study found that more than half of the respondents had a good attitude towards e-learning. This finding collaborates with some cross-sectional studies conducted in tertiary institutions in Nigeria, where four-fifths of the respondents had a favourable attitude and agreed that online education is the alternative measure for conversational in-class teaching and learning for future occurrences of any pandemic [19, 24]. The result is also supported by the findings of Pingle, who reported that undergraduates in India have a higher acceptance level of comfort working with computers and other e-learning packages than in the traditional face-to-face classroom [25]. As revealed from the findings, there may be a link between attitude and the perceived usefulness of the technology, as most students agreed that e-learning could improve the quality of learning and satisfaction with training. Thus, the high perceived usefulness positively influences students’ attitudes towards using e-learning systems. This position is congruent with the findings of an earlier study, which suggest that the most critical belief underlying an individual’s attitude towards adopting a new technology depends on the person’s perceptions of the usefulness of the technology [19]. To improve students’ attitudes towards e-learning, training and information sessions on e-learning need to focus primarily on how the technology can help improve the efficiency, productivity, and effectiveness of students’ learning process rather than on the actual use of the technology.

On students’ readiness for online medical education, the study revealed that about half of the respondents expressed willingness for a combination of in-class and e-learning approaches, while a minor two percent preferred reliance on e-learning methods only. The findings agree with a study where willingness could have been better due to reported minimal online teacher-student interaction [26]. The extent of readiness may be related to the numerous barriers to online access reported by the respondents, which is common in developing countries like Nigeria.

The findings show a significant relationship between previous experience with e-learning and readiness to use an e-learning system. Those who participated in webinars during the COVID-19 pandemic were twice more ready to adopt e-learning than those who did not. The finding could be explained by the fact that users’ prior experience with technology affects their views about technology in general. Users or potential adopters of the system would be ready to engage in the behaviour because of previous hands-on expertise. This assertion resonates with the student’s view on training in e-learning content development. However, our findings contradict a study in Nigeria, which showed no significant relationship between the level of computer experience and intention to use e-learning systems [19]. In addition, our findings show that students with good attitudes towards using the e-learning system are three times more likely to be ready for e-learning than those with a poor attitude. Attitude indicates, to a certain degree, the possibility of adopting certain behaviours, and students’ favourable and positive attitude towards an e-learning system suggests a greater probability that they will accept it [19].

### Strengths

The main strength of this study is its focus on user characteristics which influence the adoption of e-learning systems, considering that it is a prerequisite to introducing successful e-learning systems [16]

### Study limitations

The COVID-19 pandemic adversely affected medical education in Nigeria, with the closure of all higher institutions for one year to curtail the virus’s spread. However, this closure may have affected the students differently. This study took place about seven months after the medical school was re-opened. Thus, students’ responses may have been influenced by the various forms affected by the closure. Also, the participation of the first-year students in the study was limited. This was due to their involvement in the university registration formalities, which were delayed because of the university’s closure at the pandemic’s peak. In all, the study provided insight into the readiness of medical students to commence online medical education.

## Conclusion

The study concludes that most students were ready and had a good attitude towards online medical education. However, lessons from the COVID-19 pandemic reveal lapses and gaps in adopting online medical education. These barriers to e-learning range from poor internet connectivity, poor e-learning infrastructure, unavailability of personal laptops, high internet subscription costs, and lack of functional virtual library in the university, and need to be addressed.

To improve the attitude and readiness of students for online medical education, the Nigerian government should put in place measures that ensure continuity, inclusion and equity for all learners and mitigate learning disruption during this pandemic and beyond. For example, the government should invest in providing solar-powered educational gadgets pre-loaded with offline academic resources to learners and other e-learning infrastructure, including steady internet services within the confines of the university. In addition, University authorities should ensure that every enrolled medical student owns or have access to a dedicated laptop through a university-mediated arrangement. Furthermore, the institution’s authorities should implement staff training and capacity building on e-learning. To benefit from the advantages of e-learning, such as wide coverage, cost-effectiveness, uniformity, fast teaching and learning process, and rapid economic development through e-commerce, compliance with e-learning in tertiary institutions should go beyond the COVID-19 lockdown period. Thus, to facilitate students’ readiness and positive attitudes and actual usage of e-learning systems, the universities should ensure the supply of suitable support in terms of technological, economic, and organizational issues aimed at guaranteeing a high level of education to their students.

## Supporting information

S1 Dataset (SAV)

S1 File (DOCX)
