# Supplementary material for: Readiness, barriers, and attitude of students towards online medical education amidst COVID-19 pandemic: A study among medical students of Ebonyi State University Abakaliki, Nigeria
Source: PLoS One. 2023 Apr 27;18(4):e0284980. doi: 10.1371/journal.pone.0284980 (PMC10138474; doi:10.1371/journal.pone.0284980)
Supplement: S1 File — (DOCX) [file pone.0284980.s002.docx]

**Readiness, barriers and attitude of students towards online medical education during COVID-19 pandemic: a study among medical students of Ebonyi State University Abakaliki, Nigeria**

**Questionnaire**

1. Academic level ………………………………….
2. 100 level ( ) D. 200 level ( )
3. 300 level ( ) E. 400 level ( )
4. 500 level ( ) F. 600 level ( )
5. Age ……………………………………….
6. Gender A. Male ( ) B. Female ( )
7. Ethnic group
8. Igbo ( ) B. Yoruba ( )

C. Hausa ( ) D. Ijaw ( )

E. Others, specify …………………..

1. Marital status A. Single ( ) B. Married ( )
2. Religion A. Christianity ( ) B. Islam ( ) C. Traditional religion ( )
3. Father’s highest Education
4. No formal education ( ) B. Primary education ( )

C Secondary education ( ) D. Tertiary education ( )

1. Mother’s highest Education
2. No formal education ( ) B. Primary education ( )

C Secondary education ( ) D. Tertiary education ( )

1. Employment status of Father
2. Unemployed ( ) B. Self-employed C. Salaried employment
3. Employment status of Mother
4. Unemployed ( ) B. Self-employed C. Salaried employment
5. Is your Father or Mother a Medical Doctor
6. Yes ( ) B. No C. Don’t know
7. How do you rate your academic performance at present?
8. Excellent ( ) B. Good ( ) C. Fair ( )

D. Poor ( ) E. Don’t know ( )

1. Indicate the name of town/community where you are now

………………………………………………………..

1. From the information above, tick below)
2. Urban ( ) B. Rural ( )
3. Average monthly expenditure in **Naira** while in school before COVID-19 pandemic ………………………….
4. Preferred resource/source for studying and referencing in medical school before COVID-19 pandemic **(One answer)**
5. Textbooks (Hard copies) ( ) C. E-text books ( ) E. Lecture notes ( )
6. Internet ( ) D. Interaction with other students ( )
7. Reason for preferring the Internet before COVID-19 pandemic **(For those who prefer Internet as preferred resource) ………………………………………………………**
8. Commonly used websites. **Multiple responses encouraged**

a). Facebook ( ) b). WhatsApp ( ) c). Twitter ( ) d). You Tube ( )

e). Google ( ) f). Google scholar ( ) g). Instagram ( ) Yahoo ( )

1. Which of the following school-based activities is done using the internet before COVID-19 pandemic. **Tick all that apply**
2. Payment of school fees ( ) E. Submission of assignments ( )
3. Research/projects ( ) F. Further reading after lectures ( )
4. Course registration ( ) G. Lectures ( )
5. Registration for examinations ( ) H. Checking results after examination ( )

**Readiness for E-learning**

1. Do you have a personal laptop?
2. Yes ( ) B. No ( )
3. Is your laptop functional?
4. Yes ( ) B. No ( )
5. Do you have any of these devices? **Tick 1 for Yes and 0 for No**
6. Android phone ( ) B. i phone ( ) C. Tablet ( ) D. Internet modem ( )
7. Do you have a functional e-mail address?
8. Yes ( ) B. No ( ) C. Don’t know ( )
9. Do you have access to internet?

A. Yes ( ) B. No ( ) C. Don’t know ( )

23. How do you rate your current internet connectivity? (where you are now)

A. Very Good ( ) B. Good ( ) C. Fair ( ) D. Poor ( ) E. Very Poor ( ) G. Don’t know ( )

1. How much in Naira do you spend on internet services on the average in a month before the COVID-19 pandemic? ………………………………………………………..
2. How much could you spend on the maximum for internet services in a month if the school adopts e-learning program?

……………………………………………………………………

1. Do you have any previous experience with e-learning before COVID-19 pandemic?
2. Yes ( ) B. No ( ) C. Don’t know ( )
3. If Yes, which platform did you use ……………………………………………..
4. In view of COVID-19 pandemic, what do you advise the university authorities.? **CHOOSE ONE RESPONSE**
5. Resume normal lectures immediately
6. Resume normal lectures only when pandemic is over
7. Initiate e-learning platforms for the students meanwhile
8. Combine both in-class and e-learning techniques
9. Rely on only e-learning techniques for medical education
10. Does the university have a functional virtual library?
11. Yes ( ) B. No ( ) C. Don’t know ( )
12. Attitude towards IT based medical education among students

**To what extent do you agree with the following statements**

| **Variable** | **Strongly disagree** | **Disagree** | **Uncertain** | **Agree** | **Strongly agree** |
| --- | --- | --- | --- | --- | --- |
| There should be e-mail based medical education and discussion in the medical school |  |  |  |  |  |
| University should initiate Live lectures for medical teaching via ZOOM etc |  |  |  |  |  |
| Lectures for medical teaching should be made available on University e-learning portal/website where students can assess it |  |  |  |  |  |
| Hard copies of lectures **only** should be used for medical teaching |  |  |  |  |  |
| Acquisition of laptops should be made compulsory for medical students |  |  |  |  |  |
| I support the use of computer for delivering medical services to the people |  |  |  |  |  |
| Computer should be used to supplement in-class teaching in medical school |  |  |  |  |  |
| There is No place for use of computer in medical teaching |  |  |  |  |  |
| I have now made a Personal commitment to e-learning while in medical school |  |  |  |  |  |
| Use of Technology is the readiness factor for e-learning |  |  |  |  |  |
| There is Need for training of students on e-learning content development |  |  |  |  |  |
| Adopting e-learning could increase the satisfaction of medical students to their training |  |  |  |  |  |
| E-learning could improve quality of learning in a medical school |  |  |  |  |  |
| There is need to improve E-learning infrastructure in my medical school? |  |  |  |  |  |

1. Do you accept the immediate migration to e-learning as option for continued learning during this COVID-19 pandemic?
2. Yes ( ) B. No ( ) C. Don’t know ( )
3. What is the **main barrier/challenge** to commencement of E-learning in your university? **Tick only one answer**
4. There will be a high demand on time
5. Lack of technical skills on part of the students
6. Lack of technical skills on part of the lecturers
7. Cost of internet services
8. Poor network for internet connectivity
9. It will reduce one on one interaction with medical teachers
10. It may not fully prepare the students for medical examinations
11. It may not fully prepare the students for medical practice after graduation
12. Most students do not have laptops
13. The university does not have the needed infrastructure for the commencement of E-learning
